# Supplementary figures and images for: Ancient genetic divergence in bumblebee catfish of the genus Pseudopimelodus (Pseudopimelodidae: Siluriformes) from northwestern South America
Source: PeerJ. 2020 May 29;8:e9028. doi: 10.7717/peerj.9028 (PMC7265895; doi:10.7717/peerj.9028)

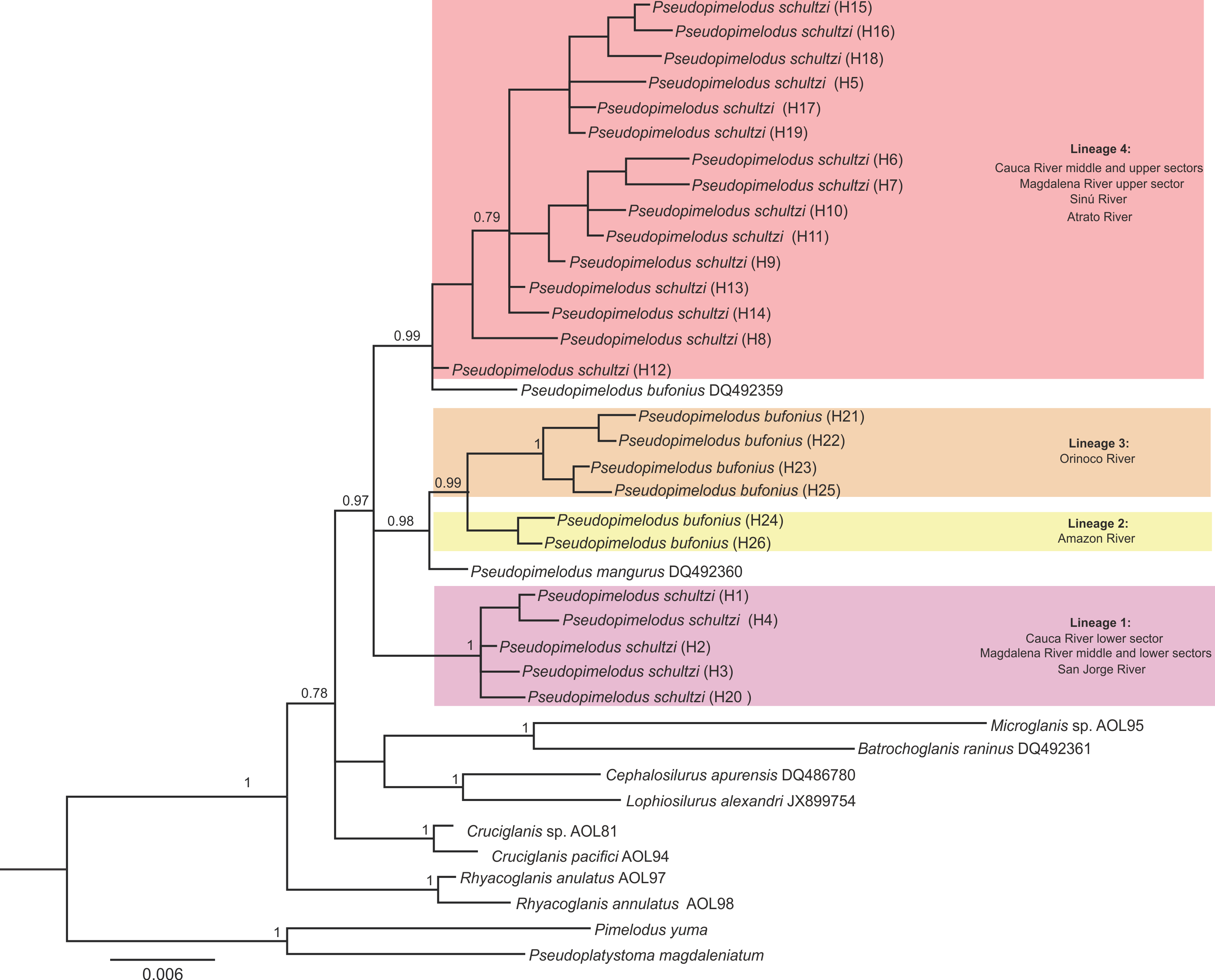

Supplement: Figure S1 — Colors denote different lineages of Pseudopimelodus. Haplotype is indicated in parenthesis. [file peerj-08-9028-s003.png]

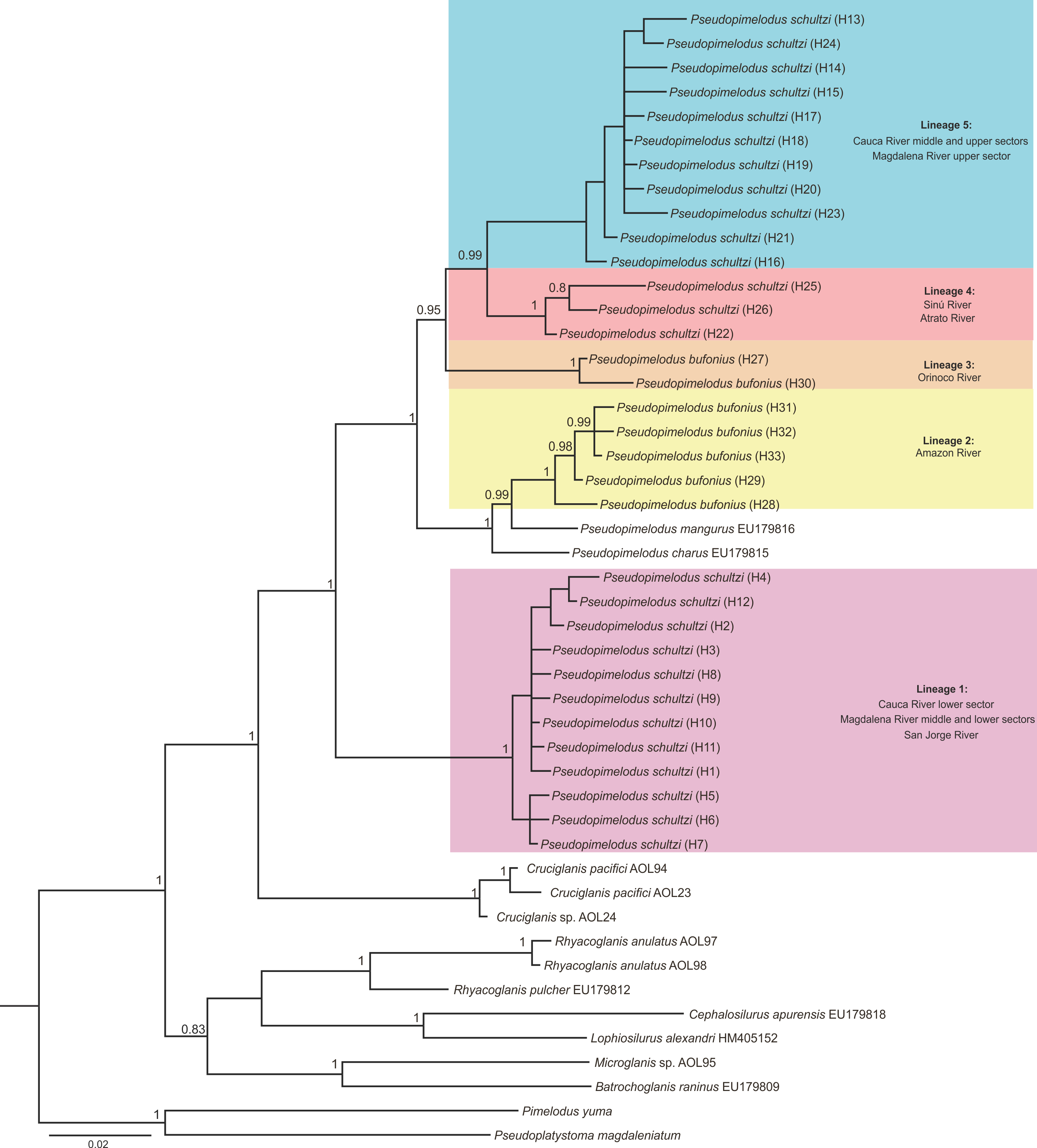

Supplement: Figure S2 — Colors denote different lineages of Pseudopimelodus. Haplotype is indicated in parenthesis. [file peerj-08-9028-s004.png]

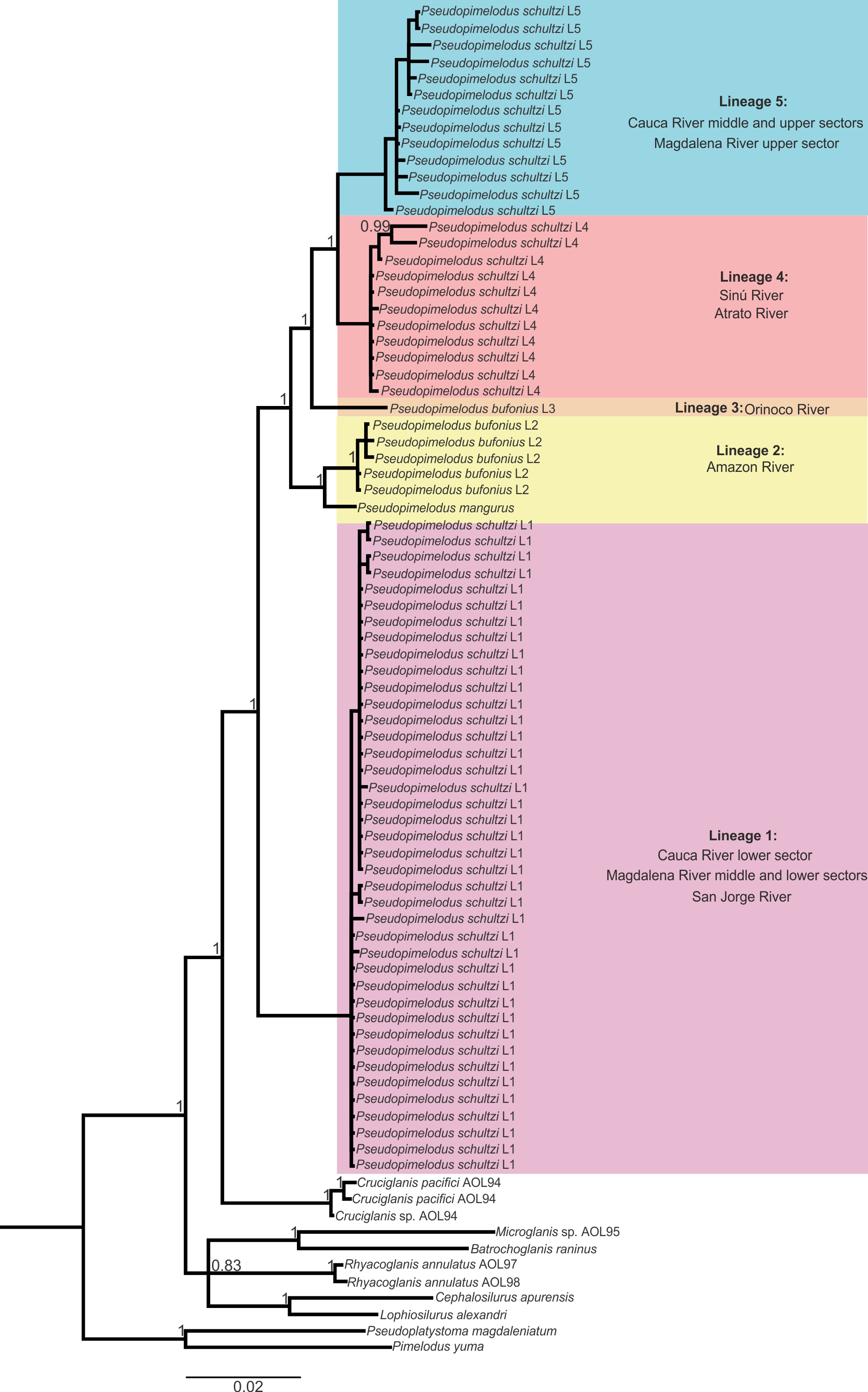

Supplement: Figure S3 — Colors denote different lineages of Pseudopimelodus. [file peerj-08-9028-s005.png]
